# Transcriptome analysis of maca (*Lepidium meyenii*) root at different developmental stages

**DOI:** 10.1002/aps3.1206

**Published:** 2018-12-17

**Authors:** Rui‐Guang Shang, Pu Yang, Bing‐Yi Wang, Zun‐Ling Zhao

**Affiliations:** ^1^ Research Institute of Resource Insects Chinese Academy of Forestry Kunming 650224 Yunnan People's Republic of China

**Keywords:** Brassicaceae, differentially expressed genes, *Lepidium meyenii*, maca, RNA‐Seq, secondary metabolite biosynthesis, transcriptome

## Abstract

**Premise of the Study:**

Maca (*Lepidium meyenii*; Brassicaceae) has been cultivated by Andeans for thousands of years as a food source and has been used for medicinal purposes. However, little is known about the mechanism underlying material accumulation during plant growth.

**Methods:**

RNA‐Seq technology was used to compare the transcriptome of black maca root at three developmental stages. Gene Ontology term enrichment analysis and Kyoto Encyclopedia of Genes and Genomes (KEGG) pathway analysis were applied for the identification of pathways in which differentially expressed genes were significantly enriched.

**Results:**

Trinity was used to de novo assemble the reads, and 120,664 unigenes were assembled. Of these, 71.53% of the unigenes were annotated based on BLAST. A total of 18,321 differentially expressed genes were observed. Gene Ontology term enrichment analysis found that the most highly represented pathway among the differentially expressed genes was for genes involved in starch and sucrose metabolism. We also found that genes involved in secondary metabolite biosynthesis, such as glucosinolate biosynthesis, were significantly enriched.

**Discussion:**

The genes that were differentially expressed between developmental time points likely reflect both developmental pathways and responses to changes in the environment. As such, the transcriptome data in this study serve as a reference for subsequent mining of genes that are involved in the synthesis of important bioactive components in maca.

*Lepidium meyenii* Walp. (maca) is a species in the Brassicaceae family. The plant is cultivated at elevations of 4000–4500 m in the central Andes (Gonzales et al., 2009). Maca has been used for centuries in the Andes as a food source (León, 1964), and in recent years there has been increasing interest in its medicinal properties. Animal studies have shown that maca can improve sperm quantity and quality (Clément et al., 2010) and is beneficial for increasing endurance capacity during exercise (Choi et al., 2012). Other medicinal properties attributed to maca include the ability to reduce depression and anxiety (Gonzales et al., 2009), improve learning and memory (Rubio et al., 2011), reduce prostate size (Gonzales et al., 2005), and balance hormonal secretion (Fumiaki et al., 2014). However, the reproductive benefits of maca require more research due to a lack of comprehensive scientific studies (Beharry and Heinrich, 2018). Dini et al. (1994) found that maca root is of nutritional interest and could be used as a food source. The potential bioactive ingredients in maca contain several secondary metabolites of interest, including glucosinolates, phytosterols, alkaloids, and alkamides (Wang et al., 2007; Campos et al., 2013). However, current research on maca focuses mainly on its medicinal and pharmacological properties; systematic metabolomic and molecular studies are lacking. The maca genome has been published (Zhang et al., 2016), but to the best of our knowledge, there are no previous reports that utilize RNA‐Seq to characterize the transcriptional changes during maca root development.

RNA‐Seq approaches are now commonly used for transcriptome analysis and have been conducted in a wide range of plant and animal species (Guo et al., 2014; Garcia‐Seco et al., 2015; Maboreke et al., 2016; Somvanshi et al., 2016; Wippler et al., 2016). Given the versatility of transcriptome analysis, it is possible to gain insight into developmental pathways (intrinsic changes) or analyze responses to changes in environmental conditions (extrinsic changes). When RNA‐Seq is coupled with term enrichment analysis (e.g., Gene Ontology [GO] terms), one can identify biological processes that are active or suppressed under certain conditions, such as metabolic or signaling pathways (Yang et al., 2015). In this study, we used genome‐wide transcript profiling to predict the accumulation of compounds and metabolites during the development of maca root. Because the biosynthesis and accumulation of certain secondary metabolites are often stage‐ and tissue‐specific (Salam et al., 2014; Yu et al., 2015), we analyzed several development stages of maca root, which we believe provides an important foundation for the identification of candidate genes acting in the mechanism underlying material accumulation during root growth.

## MATERIALS AND METHODS

Black maca roots grown in Dahai, Huize County, Yunnan Province, China, were used in this study. From August 2012 to February 2013, we harvested 100 maca root specimens (from 100 individuals) every month to investigate the growth dynamics of root diameter and root fresh weight. The results showed that the growth curve of maca root was S‐shaped (Appendix S1). Based on these findings, we divided the development of maca root into three stages: an early stage with slow growth (stage I), a middle stage with fast growth (stage II), and a late stage when growth slows and then stops (stage III). We then harvested maca roots in September 2015 (stage I), November 2015 (stage II), and January 2016 (stage III). For the RNA‐Seq samples, we harvested 15 specimens (from 15 individuals) from each time point, which were randomly divided into three replicate groups, and the epidermis was quickly removed. The remaining tissue of each specimen was then cut into small pieces and stored in liquid nitrogen until later usage. Each group was considered as one biological repetition. Total RNA was isolated using TRIzol Reagent (Invitrogen, Carlsbad, California, USA) according to the manufacturer's instructions. DNase Ι (TaKaRa Bio Inc., Otsu, Shiga, Japan) was added to remove any genomic DNA contamination in the total RNA. Finally, the integrity and concentration of the RNA were measured using the Agilent 2100 Bioanalyzer system (Agilent Technologies, Santa Clara, California, USA).

We then isolated mRNA via oligo(dT) magnetic beads (Thermo Fisher Scientific, Waltham, Massachusetts, USA), followed by fragmentation using the Covaris M220 Focused‐ultrasonicator (Covaris, Woburn, Massachusetts, USA), allowing for ~200 bp average length. The mRNA fragments were used as a template to generate first‐strand cDNAs via random hexamer‐primers, followed by second‐strand cDNA synthesis. cDNA preparations were purified via the Agencourt AMPure XP kit (Beckman Coulter, Brea, California, USA). Adapters were ligated to the cDNA fragments after generating blunt‐ends and 3′‐addition of polyA tails. The fragments were then amplified by PCR. Strand‐specific library preparation and paired‐end sequencing (read length 125 nucleotides) were performed on the Illumina HiSeq 2500 platform (Illumina, San Diego, California, USA) by Berry Genomics (Beijing, China). Raw sequence reads were filtered using Trimmomatic version 0.30 (Bolger et al., 2014) to remove nonsense sequences including adapters, low‐quality reads, reads containing poly‐N, and other very short sequences. Clean reads were pooled to assemble a reference transcriptome with the Trinity software package (version 2.1.1) using the default parameters (Grabherr et al., 2011).

Unigenes were annotated via BLAST (Götz et al., 2008) by mining the following databases using an *E*‐value cutoff of 10^−5^: the National Center for Biotechnology Information (NCBI) non‐redundant protein sequence database (nr), the NCBI non‐redundant nucleotide (nt) database, the Gene Ontology (GO) database, the Kyoto Encyclopedia of Genes and Genomes (KEGG) database, the euKaryotic Orthologous Groups (KOG) database, the Pfam database (a large collection of protein families), and SwissProt. Gene expression levels are based on FPKM (fragments per kilobase of transcript per million mapped reads). The quantification of fold changes and analysis of differential gene expression was carried out with Cufflink (Trapnell et al., 2012). The software edgeR was used to analyze the differential expression genes (Robinson et al., 2010). Differentially expressed genes between two samples were defined as having a log_2_ ratio ≥1 and a false discovery rate (FDR) ≤0.001. Term enrichment analyses were carried out for GO and KEGG terms.

## RESULTS

### De novo transcriptome sequencing of maca root

To characterize the transcriptome of maca root and to generate expression profiles, we collected samples representing the three developmental stages of the plant as defined above, and sequenced corresponding cDNA samples using the Illumina HiSeq 2500. More than 162 million raw reads were generated from all three developmental stages, amounting to ~23.8 Gbp of data. The raw reads were submitted to the NCBI Sequence Read Archive (BioProject ID PRJNA450202, SRA accession SRP139986). The Transcriptome Shotgun Assembly project has been deposited at GenBank (accession GGNI00000000), and the version described in this paper is the first version (GGNI01000000). The de novo assembled transcriptome had 182,270 transcripts. The average GC content of the maca root transcriptome was 39.7%. The longest transcript of each gene was used as a unigene, and de novo assembly of the filtered reads was assembled into 120,664 unigenes. Transcript and unigene length ranged from 200 to >5000 bp.

### Annotation and classification of gene function

**Figure 1:**
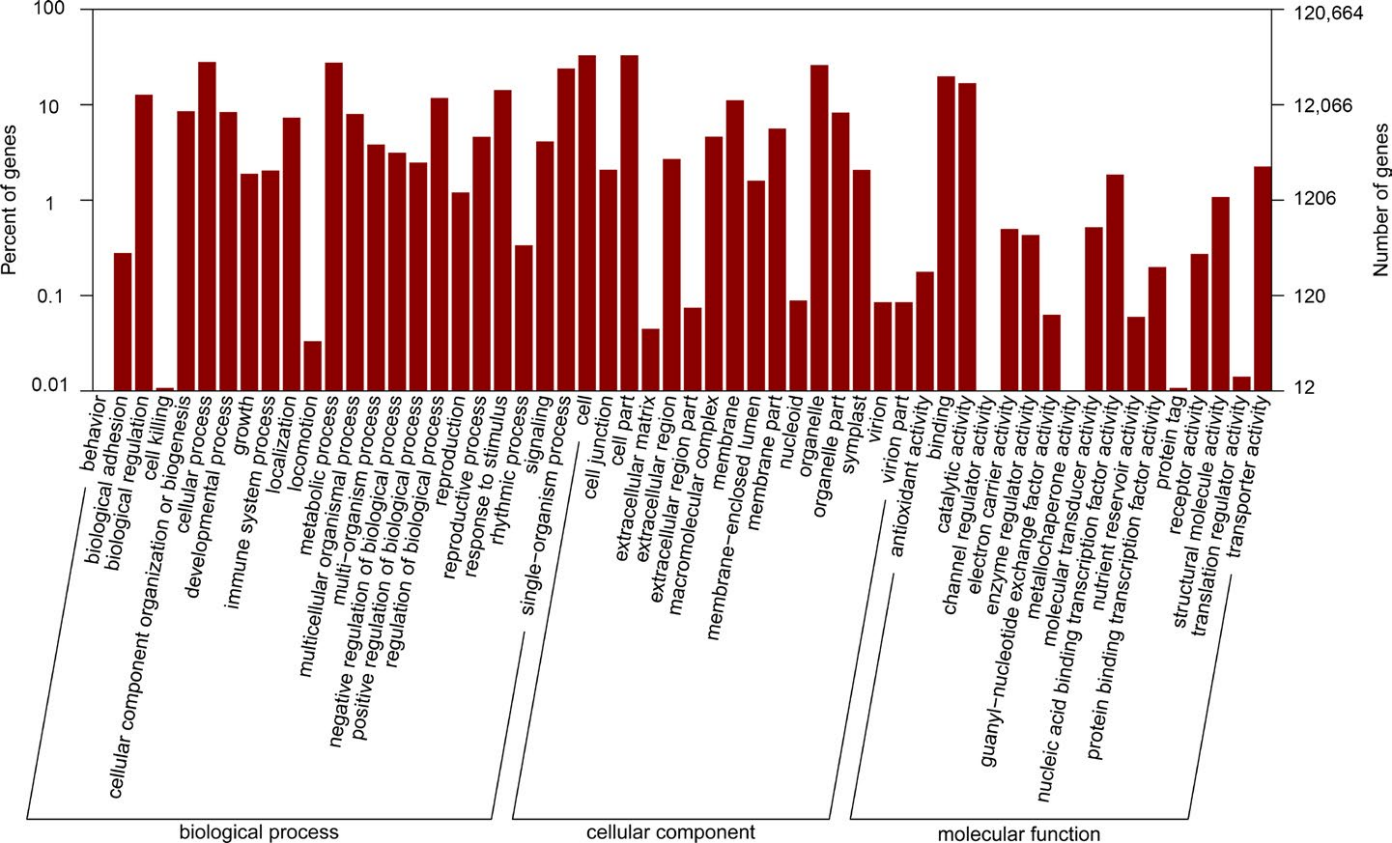
Histogram of all‐unigene Gene Ontology (GO) database classification of maca root consensus sequences. Results are summarized for the three main GO categories: biological process, cellular component, and molecular function. The left axis indicates the percent of sequences in each category, and the right axis shows the total number of genes in each category.

**Figure 2:**
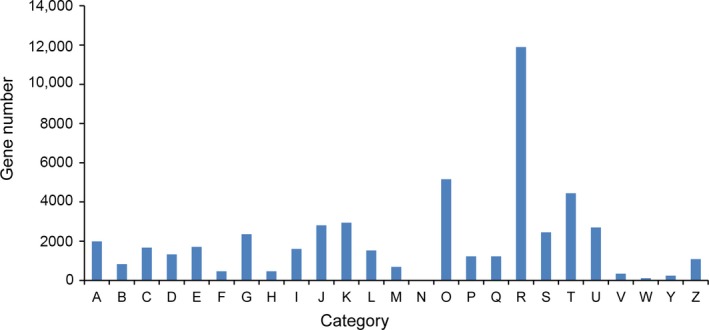
euKaryotic Orthologous Groups (KOG) classification of maca root all‐unigenes. A = RNA processing and modification; B = chromatin structure and dynamics; C = energy production and conversion; D = cell cycle control, cell division, chromosome partitioning; E = amino acid transport and metabolism; F = nucleotide transport and metabolism; G = carbohydrate transport and metabolism; I = lipid transport and metabolism; J = translation, ribosomal structure, and biogenesis; K = transcription; L = replication, recombination, and repair; M = cell wall/membrane/envelope biogenesis; N = cell motility; O = posttranslational modification, protein turnover, chaperones; P = inorganic ion transport and metabolism; Q = secondary metabolites biosynthesis, transport, and catabolism; R = general function prediction only; S = function unknown; T = signal transduction mechanisms; U = intracellular trafficking, secretion, and vesicular transport; V = defense mechanisms; W = extracellular structures; Y = nuclear structure; Z = cytoskeleton.

**Figure 3:**
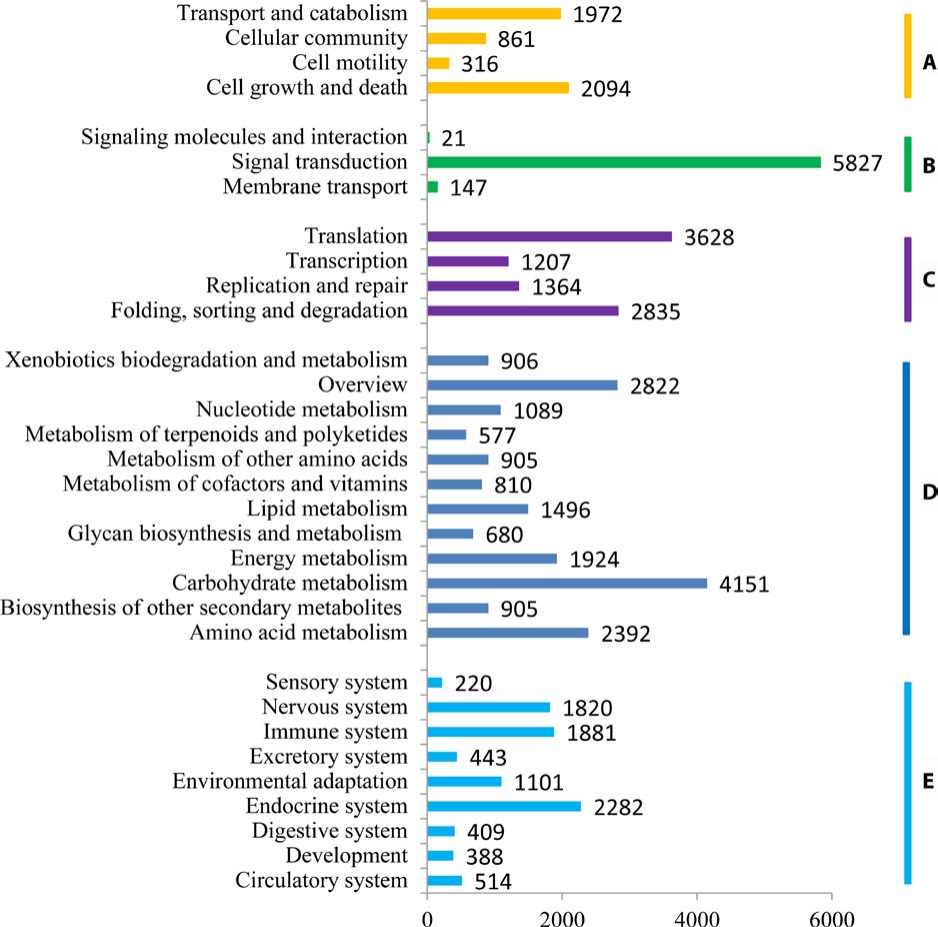
Kyoto Encyclopedia of Genes and Genomes (KEGG) classification of maca root all‐unigenes. A = cellular processes; B = environmental information processing; C = genetic information processing; D = metabolism; E = organismal systems.

Through analysis of GO databases, 50,303 unigenes were assigned to 56 categories (Fig. 1). A total of 46,237 unigenes were categorized into 25 groups by the KOG database (Fig. 2). “General functional prediction only” (“R” in Fig. 2) represented the major group, followed by “posttranslational modification, protein turnover, chaperones” (“O” in Fig. 2), and “signal transduction mechanisms” (“T” in Fig. 2). In this study, 30,539 unigenes were classified with the KEGG database. Among these, 9058 unigenes (7.5% of the total) were assigned to “organismal systems,” 18,657 (15.46%) to “metabolism,” 9034 (7.49%) to “genetic information processing,” 5995 (4.97%) to “environmental information processing,” and 5243 (4.35%) to “cellular processes” (Fig. 3). To optimize our efforts to functionally annotate all unigenes, 72,181, 70,191, 19,238, and 47,396 consensus sequences were annotated using the nr, nt, Pfam, and SwissProt databases, respectively (Table 1). Generally, a total of 71.53% *L. meyenii* unigenes were annotated, and the remaining 28.47% unigenes could not be annotated because of low or absent similarity to the sequences in the seven public databases.

**Table 1:** Unigene functional annotation statistical results

Database	No. of annotated sequences^a^	Percentage of all unigenes (%)
GO	50,303	41.69
KEGG	30,539	25.31
KOG	46,237	38.32
nr	72,181	59.82
nt	70,191	58.17
Pfam	19,238	15.94
SwissProt	47,396	39.28
Annotated	86,310	71.53
Total unigenes	120,664	100.00

*Note:* GO = Gene Ontology database; KEGG = Kyoto Encyclopedia of Genes and Genomes database; KOG = euKaryotic Orthologous Groups database; nr = NCBI non‐redundant protein sequence database; nt = NCBI non‐redundant nucleotide database.

a *E*‐value < 10^−5^.

**Figure 4:**
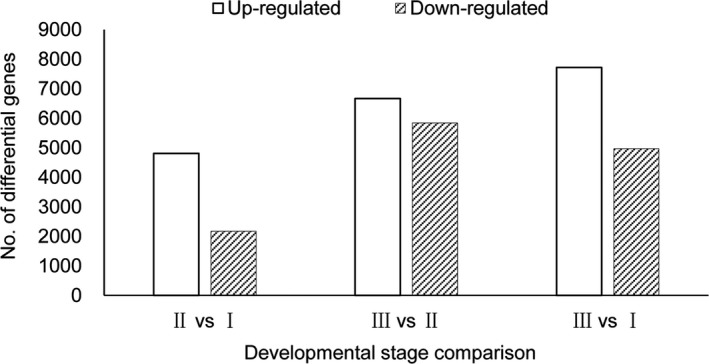
Number of differentially expressed genes between three developmental stages in maca root. The statistical threshold was log_2_ ratio ≥ 1.

We screened genes for significant differential expression between the three defined root stages. There were 6975 differentially expressed genes identified between stage III and stage I, with 4804 genes that were up‐regulated and 2171 genes that were down‐regulated. Between stage III and stage II, we identified 12,508 differentially expressed genes, of which 6669 genes were up‐regulated and 5839 genes were down‐regulated. Finally, 12,682 differentially expressed genes were identified between stage III and stage I, with 7717 genes that were up‐regulated and 4965 genes that were down‐regulated. A comparison between the differentially expressed genes among the three stages is shown in Figure 4.

### Analysis of differentially expressed genes

**Figure 5:**
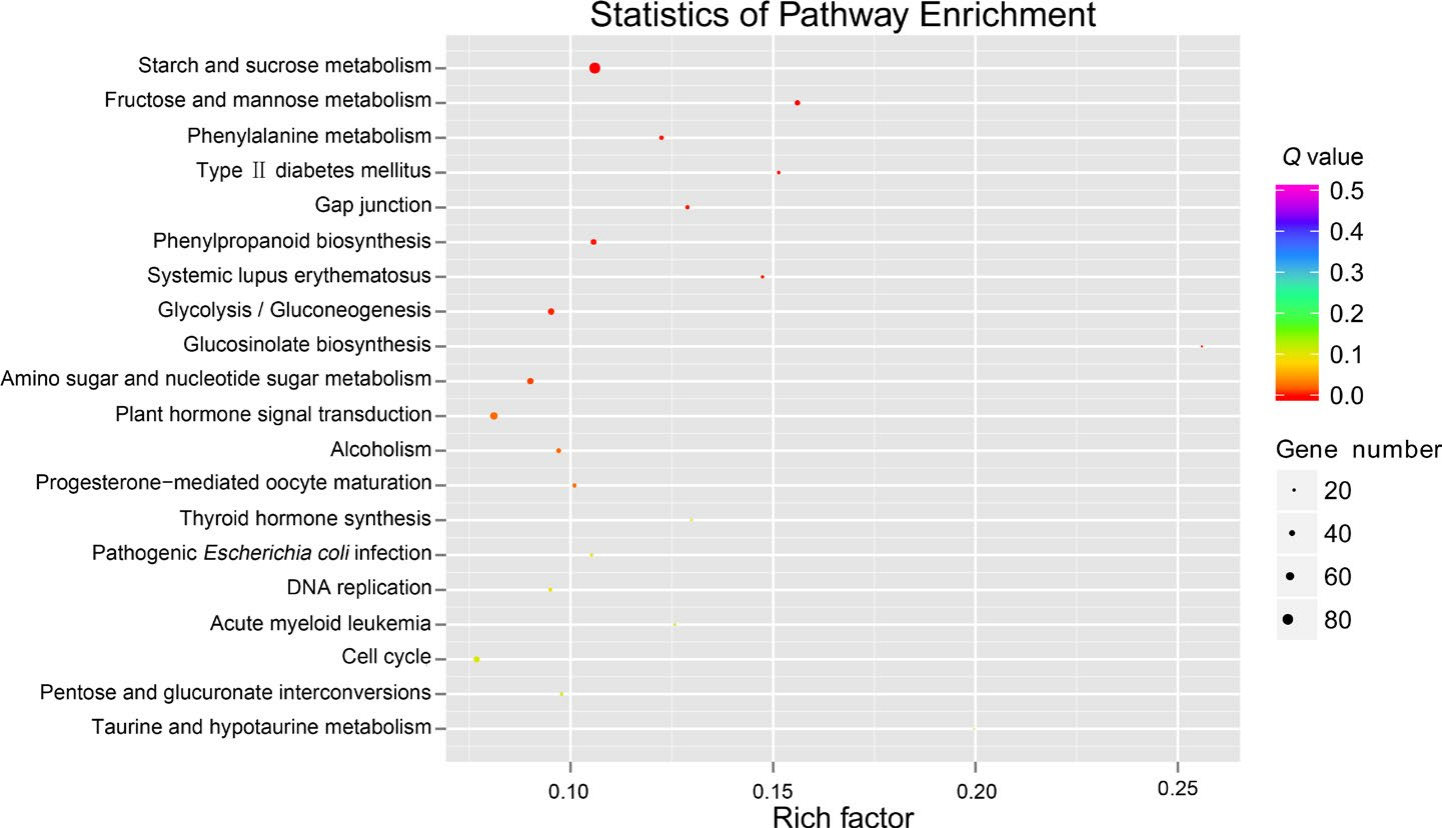
Scatter diagram of the top 20 Kyoto Encyclopedia of Genes and Genomes (KEGG) pathway enrichment terms based on differentially expressed genes. The rich factors indicate the ratio of the number of differentially expressed genes mapped to a certain pathway to the total number of genes mapped to this pathway. Greater rich factor means greater intensiveness. The *Q* value was calculated using the hypergeometric test through Bonferroni correction. *Q* value is corrected *P* value ranging from 0–1, and a smaller *Q* value signifies greater intensiveness. Gene number means number of differentially expressed genes mapped to a certain pathway.

We performed term enrichment analyses for both GO and KEGG terms to characterize differentially expressed genes that may have potential roles in metabolic pathways. GO enrichment classification was carried out using the GOseq package (Guo et al., 2014). We considered GO terms to be significantly enriched when the corrected *P* value was <0.05. In summary, 9967 differentially expressed genes were classified into “biological process,” 4296 genes were classified into “cellular component,” and 4058 genes were classified into “molecular function.” The majority of genes involved in root growth and development were strongly enriched, such as cell proliferation (GO:0008283), cell wall organization (GO:0071555), and xyloglucan:xyloglucosyl transferase activity (GO:0016762). For term enrichment analysis of differentially expressed genes based on KEGG pathways, we used the KOBAS software package (Guo et al., 2014). We mapped differentially expressed genes to 288 KEGG pathways, and the most highly represented pathway was starch and sucrose metabolism (Ko00500) (Fig. 5). Starch is the major carbohydrate of tuber and root crops (Hoover, 2001); sucrose and other sugars serve as substrates for starch production, and they also play a regulatory role in root development and various metabolic processes (Ravi et al., 2009). The larger part of maca root is formed by parenchyma, which is rich in starch and sugar (León, 1964). Dried maca root contains 59% carbohydrates and 8.5% fiber (Dini et al., 1994). The results from the term enrichment analysis suggest that during development maca root undergoes rapid cell division and proliferation followed by mass production of carbohydrates.

It is interesting to note that we also found that GO terms corresponding to stress responses, such as cold (GO:0009409) and wounding (GO:0009611), were significantly enriched. This may be caused by a gradual decrease in temperature starting in October, which explains the enrichment of cold‐response GO terms as the maca root adapts to increasingly colder temperatures. It is also worth noting that some genes involved in secondary metabolite biosynthesis were significantly enriched, as they were associated with the terms “positive regulation of flavonoid biosynthesis” (GO:0009963) and “glucosinolate biosynthetic process” (GO:0019761). Additionally, glucosinolate biosynthesis (PATHWAY: ko00966) was one of the top 20 KEGG pathway enrichment terms (Fig. 5). This result is consistent with the analysis of the maca genome (Zhang et al., 2016), which showed that gene families involved in secondary metabolite biosynthesis (i.e., glucosinolate biosynthesis) underwent expansion in this species. Glucosinolates and their derivatives have attracted much attention because of their biological activities. For example, benzyl isothiocyanate is a potent inhibitor of breast cancer, stomach cancer, and liver cancer (Wattenberg, 1981; Rosa et al., 2010).

Maca root has a comparatively high concentration of glucosinolates, and the glucosinolate content of fresh maca root is approximately 100 times higher than that in other cruciferous crops such as cauliflower and cabbage (Li et al., 2001). Studies in animals suggest that maca root has a range of pharmacological functions, such as enhancing fatigue resistance (Ikeuchi et al., 2009), anticancer activity (Večeřa et al., 2007), and reducing prostate hyperplasia (Gonzales et al., 2008). These reported pharmacological activities were attributed to the high glucosinolate concentrations present in maca root. Therefore, it is necessary to further study the metabolism of glucosinolate biosynthesis and to use genomics to identify some of the key genes involved in its regulation.

## CONCLUSIONS

A total of 182,270 transcripts and 120,664 unigenes were identified from three developmental stages of black maca root using RNA‐Seq and de novo assembly via the Trinity software package. A total of 18,321 genes were identified as differentially expressed in these three developmental stages. These genes fall into distinct molecular pathways and are closely related to regulating root development and secondary metabolite biosynthesis. To the best of our knowledge, this is the first study that investigated tissue‐specific transcript profiles of black maca root during different developmental stages. Our results will provide a foundation for future investigations aimed at elucidating the molecular mechanisms of compound accumulation during maca root development.

## DATA ACCESSIBILITY

The raw reads were submitted to the National Center for Biotechnology Information (NCBI) Sequence Read Archive (BioProject ID PRJNA450202, SRA accession SRP139986). The Transcriptome Shotgun Assembly project has been deposited at GenBank (accession GGNI00000000), and the version described in this paper is the first version (GGNI01000000).

## Supporting information


**Appendix S1**. The growth dynamics of diameter and fresh weight of maca root.
