# Supplementary figures and images for: Transcriptome analysis of maca (Lepidium meyenii) root at different developmental stages
Source: Appl Plant Sci. 2018 Dec 17;6(12):e01206. doi: 10.1002/aps3.1206 (PMC6303156; doi:10.1002/aps3.1206)

**Appendix S1.** The growth dynamics of diameter and fresh weight of maca root.

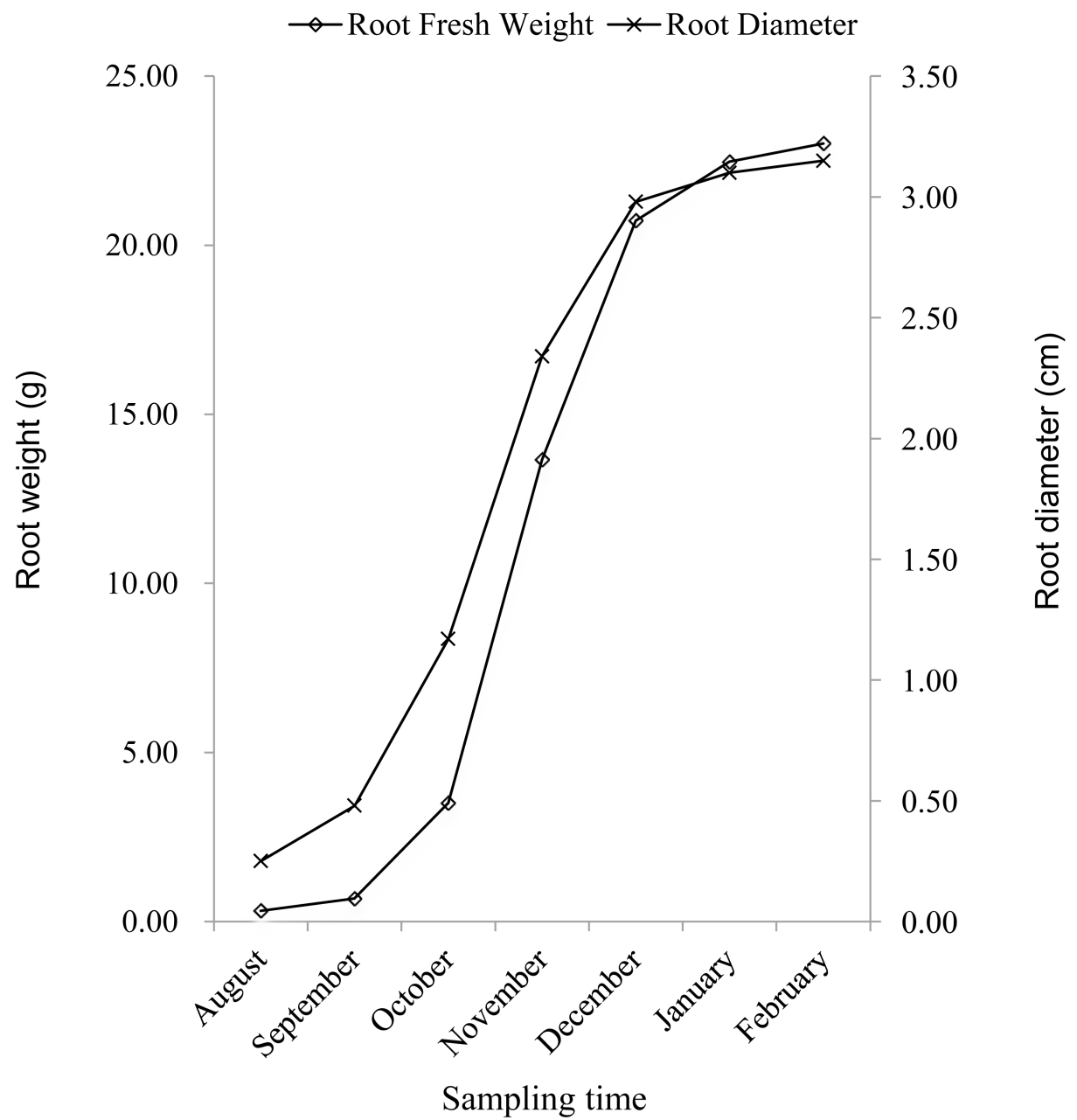

Supplement: Supplementary file 1 — Appendix S1. The growth dynamics of diameter and fresh weight of maca root. [file APS3-6-e01206-s001.pdf]
